# Knowledge, Attitude and Practice of Standard Infection Control Precautions among Health-Care Workers in a University Hospital in Qassim, Saudi Arabia: A Cross-Sectional Survey

**DOI:** 10.3390/ijerph182211831

**Published:** 2021-11-11

**Authors:** Adil Abalkhail, Mahmudul Hassan Al Imam, Yousif Mohammed Elmosaad, Mahmoud F. Jaber, Khaled Al Hosis, Fahad A. Alhumaydhi, Thamer Alslamah, Ali Alamer, Ilias Mahmud

**Affiliations:** 1 Department of Public Health, College of Public Health and Health Informatics, Qassim University, Al Bukairiyah 52741, Saudi Arabia; abalkhail@qu.edu.sa (A.A.); mf.jaber@qu.edu.sa (M.F.J.); 4037@qu.edu.sa (T.A.); 2 School of Health, Medical and Applied Sciences, Central Queensland University, Rockhampton, QLD 4701, Australia; m.alimam@cqu.edu.au; 3 Central Queensland Public Health Unit, Central Queensland Hospital and Health Service, Rockhampton, QLD 4700, Australia; 4 College of Applied Medical Sciences, King Faisal University, Al Hufuf 36362, Saudi Arabia; yelmosaad@kfu.edu.sa; 5 Department of Nursing Education, Nursing College, Qassim University, Buraydah 51452, Saudi Arabia; 3747@qu.edu.sa; 6 Department of Medical Laboratories, College of Applied Medical Sciences, Qassim University, Buraydah 51571, Saudi Arabia; f.alhumaydhi@qu.edu.sa; 7 Department of Radiology, College of Medicine, Qassim University, Buraydah 51452, Saudi Arabia; ali.alamer@qu.edu.sa

**Keywords:** KAP, hospital-acquired infections, infection control, health-care workers, Saudi Arabia

## Abstract

Hospital-acquired infections (HAIs) contribute to increased length of hospital stay, higher mortality and higher health-care costs. Prevention and control of HAIs is a critical public health concern. This study assessed the knowledge, attitude, and practice (KAP) of standard infection control precautions among health-care workers (HCWs) in Qassim, Saudi Arabia. A cross-sectional online survey among HCWs was conducted using a structured questionnaire. Predictors of KAP were investigated using multivariate logistic regression analyses and independent sample *t*-tests. A total of 213 HCWs participated in the survey. The prevalence of good (≥80% correct response) knowledge, attitude, and practice were 67.6%, 61.5%, and 73.2%, respectively. The predictors of good knowledge included the age of the HCWs (>34 years) (adjusted odds ratio: 30.5, *p* < 0.001), and training (13.3, *p* < 0.001). More than 6 years of work experience was a significant predictor of having a positive attitude (5.5, *p* < 0.001). While the predictors of good practice were having >6 years of experience (2.9, *p* < 0.01), previous exposure to HAIs (2.5, *p* < 0.05), and training (3.5, *p* < 0.01). However, being female (0.22, *p* < 0.001) and older (>34 years) (0.34, *p* < 0.01) were negatively associated with knowledge. Results indicate that arranging training for HCWs might be useful in improving their knowledge of standard infection control precautions and is also expected to facilitate positive attitude and practice.

## 1. Background

The burden of HAIs is on the rise globally despite advancements in medical care and technologies [1]. According to the World Health Organization (WHO), the prevalence of HAIs ranges between 5.7% and 19.1% in hospital settings globally [2]. Recent studies estimated the prevalence of HAIs in Europe [3] and the USA [4] at 6.5% and 3.2%, respectively. The burden of HAIs is strikingly higher in low-resourced countries compared with high-income countries [5,6,7]. A WHO-led systematic review revealed that the prevalence of HAIs varies between 7.6% and 15.5% in high-income and low- and middle-income countries, respectively [2]. HAIs contribute to increased length of hospital stay, high mortality, higher health-care costs, and economic burden on families, communities, and countries at large [2,8]. Hence, prevention and control of HAIs appear as a critical public health concern [9].

The contaminated hands of health-care workers (HCWs) and health-care equipment have been identified as the primary sources of HAIs [6,10]. The pathogens of HAIs are commonly transmitted from one patient to another when HCWs do not perform hand hygiene properly following caring for one patient and contacting another patient [11]. The incidence of HAIs varies in different types of clinical departments. A study in Norway reported that the greatest infection rate is in the intensive care units followed by neonatal and burns units [12].

The WHO reported that improper environmental hygiene and waste disposal procedures, poor infrastructure, inadequate equipment and manpower, overcrowding, limited knowledge and poor practices of basic infection control measures, and lack of national guidelines are the key determinants of HAIs [13]. The Center for Disease Control and Prevention (CDC) developed Standard Precautions describing detailed procedures that need to be followed to prevent the transmission of disease-causing agents and thereby preventing HAIs [14]. The standard infection control precautions warrant a uniform protocol to be always followed for all patients in all settings [1]. The principle of this guideline is that all patients carry infectious agents even when they are asymptomatic [14]. The standard precautions include hand hygiene, use of gown, cleaning and disinfection of equipment, facial protection (e.g., masks and goggles), disposal of sharp objects, management of medical waste and coughing etiquette [1]. However, Hein and colleagues [15] reported that adherence to hand hygiene recommendations among HCWs is below standard, with a 30% compliance rate in Burkina Faso. It was found that about 42% of Corona Virus Disease-2019 among HCWs is associated with improper personal protective equipment (PPE) use [16]. Hefty workload, prolonged clinical methods and skin status have been reported as key barriers in maintaining hand hygiene recommendations [17,18].

Some urban hospitals in the Kingdom of Saudi Arabia (KSA) recorded 2.2% of hospital infections monthly, and other reports confirmed that hospital infection is still one of the most common health problems in the KSA [19]. Al Ra’awji et al. [20] observed that more than one quarter (37%) of HCWs in the KSA had poor knowledge of hand hygiene and there is a high need for training for the HCWs in this country. The KSA has been trying to activate all infection control guidelines to improve the activities in the field of infection control to high standards [21].

According to WHO, poor knowledge, attitude, and practice (KAP) are among the key predictors of HAIs [13]. While narrating the KAP theory, Kelman argued that knowledge is essential to change practice and a positive attitude is a key instigator to bring change [22,23]. Therefore, assessment of KAP among the HCWs is crucial to explore the reasons for non-compliance and identify the measures that should be undertaken to improve infection control practice and prevent HAIs [11]. Our literature search revealed a few studies reporting the KAP of hand hygiene and infection control measures in the KSA. Among the published studies, one focused on the hand hygiene of HCWs [20], three studied infection control among dental students [24,25,26], one assessed infection control measures among HCWs in dental clinics [27], one investigated cross-infection and infection control in dental patients [28], one incorporated health science students to assess standard precautions and infection control [29], and one evaluated prevention and control of HAIs among HCWs and non-HCWs [21]. Al Ra’awji et al. [20] studied the KAP of hand hygiene among HCWs; however, the authors did not report practice aspects. No study has examined the KAP on standard precautions of infection control among HCWs in the KSA. In this context, we aimed to assess the status of KAP regarding infection control standard precautions among HCWs in the Qassim University Medical City, KSA.

## 2. Methods

### 2.1. Study Design and Setting

We conducted a cross-sectional survey of the HCWs in the Qassim University Medical City, KSA between November 2020 and February 2021. Qassim University Medical City is the first specialized academic medical city in the Qassim region, KSA. It has 278 HCWs including physicians of different disciplines, dentists, nurses, and pharmacists and medical technologists https://qumc.edu.sa/ (accessed on 14 September 2021).

### 2.2. Instrument

We assessed the knowledge, attitude, and practice (KAP) of standard infection control precautions using a self-administered structured questionnaire. We developed our KAP questions on infection control standard precaution based on the guidelines of the CDC [30] and WHO [31]. The questionnaire was divided into four parts: the first part included questions on demographic and professional information of the HCWs; the second, third, and fourth parts respectively focused on the knowledge, attitude, and practices regarding infection control standard precautions.

We assessed knowledge using a 20-item scale. We provided 1 point for each right answer and 0 points for each wrong answer. The maximum possible score was 20 points with a range from 0 to 20 points. The overall level of knowledge was classified as poor (<10 points, <50% right answer), moderate (10–15 points, 50–79% right answer), and good (16–20 points, 80–100% right answer). For the logistic regression analysis knowledge was recoded into two groups, good (16–20 points, 80–100% right answers) and moderate to poor (<16 points, <80% right answers).

We used 14 statements to assess HCWs’ attitude towards infection control standard precautions. Each statement was assessed on a five-point Likert-type scale (strongly disagree to strongly agree). The maximum possible score was 70 points with a range from 14 to 70 points. Attitudes were classified as poor (<35 points, <50% score), moderate (35–55 points, 50–79% score), and positive (36–70 points, 80–100% score). For the logistic regression analysis attitude was recoded into two groups, positive (56–70 points, 80–100% score) and poor to moderate (<56 points, <80% score).

We assessed the practice of infection control standard precautions using 15 questions on practicing standard precautions. Participants were given 1 point for each activity they were always practicing and 0 points for not practicing. The maximum possible score was 10 points with a range from 0 to 50 points. The overall level of practice was classified as poor (<8 points, <50% score), moderate (8–11 points, 50–79% right answer), and good (12–15 points, 80–100% score). For logistic regression, the analysis practice was recoded into two groups-good (12–15 points, 80–100% score), and moderate to poor (<12 points, <80% score).

### 2.3. Data Collection

Data were collected between 15 November 2020, and 15 February 2021. We disseminated our online questionnaire through our professional network using emails and WhatsApp. Participants were requested to avoid multi-registration. Before starting data collection, we pre-tested our online questionnaire on 20 HCWs from different facilities. Pretesting feedback was used to improve the wording of the questions and response options.

### 2.4. Statistical Analysis

Data were analyzed using the Statistical Package for the Social Sciences (IBM SPSS Statistics v.20, Armonk, NY, USA). The descriptive analyses were carried out to analyze participants’ demographic information, mean knowledge, attitude, and practice score of the HCWs. Descriptive analyses results were presented in tables reporting percentages and frequencies. To investigate the association between KAP and socio-demographic variables, we conducted a multivariable logistic regression analysis and independent sample *t*-tests. A *p*-value of <0.05 was considered statistically significant for both tests. For the multivariable logistic regression analyses, we reported the odds ratio (OR) with a 95% confidence interval (CI). To compare the mean knowledge, attitude, and practice between different socio-demographic and professional groups we performed an independent sample *t*-test. For these tests, we reported mean knowledge, attitude, and practice scores of different groups and reported mean differences with 95% CI.

## 3. Results

A total of 213 HCWs participated in the survey out of a total of 278 HCWs in the Qassim university medical city with a response rate of 76.62%. Among them, 67.1% were 30–34 years of age; 56.3% were males; 67.6% had more than 6 years of experience as an HCW; 30.5% were previously exposed to infection while working and 84.5% received training in infection control practices (Table 1).

Table 2 depicts the number of the participants with the correct responses in each knowledge statement. We found that 67.6% of the HCWs had good knowledge (≥80% correct response) about infection control standard precautions. The mean (±SD) score for knowledge was 15.7 (±2.7) with a range from 9 to 20.

We found that majority of the HCWs correctly responded to the knowledge statements related to using standard precautions for all patients regardless of their diagnosis and perceived infection status (96.2%) and isolation precaution (76.1%), performing hand hygiene after contact with the patient’s environment (95.8%), before and after patient care (96.2%), and before and after handling potentially infectious materials (93.9%). Moreover, they correctly responded to the knowledge statements related to wearing (98.1%) and changing gloves (95.8%) for each patient; using surgical masks (86.1%) and gowns or aprons (85.4%) to block contaminants and segregation of clinical and non-clinical waste for preventing the spread of infection (96.2%). All participants stated that a mask must be placed on coughing patients to prevent the potential dissemination of infectious respiratory secretions from the patient to others. While fewer participants correctly responded to the statements related to linen from an infectious patient (45.5%), throwing of ampoules injection that has been used in the clinical waste bin (37.1%), and only 10.3% of them were considered stationary, telephones kept in wards, and doorknobs as sources of infections.

The results presented in Table 3 showed that 61.5% of study participants had positive attitudes towards infection control standard precautions. The mean (±SD) score for attitudes was (55.5 ± 5.7) with a range of 35–65. The Majority of the HCWs had a positive attitude towards performing hand hygiene before and after any intervention with patients (91.5%), the effectiveness of standard precautions in preventing the spread of infections (87.8%), ensuring the adequate disinfection of medical equipment (85.0%), reducing the transmission of infectious organisms by adhering to standard and contact precautions (78.4%) and considered stationeries, telephones, and doorknobs are sources of infections (74.2%).

However, only 38.5%, 16%, and 12.7% strongly disagreed with the statements that standard precautions are not easy to follow, it is not logical to assume all patients are contagious unless their infection has been confirmed and it is difficult to work wearing PPE (Table 3).

Regarding practices of the HCWs’ infection control standard precautions, Table 4 shows that 73.3% of participants had good practice of infection control standard precaution (≥80% score). The mean practice score (14.2) was closer to the maximum attainable score (15). Among the participants, 84.4% reported always wearing gloves when handling saliva or sputum culture; 82.2% reported always wearing a mask when performing operations/procedures that might induce spraying of blood, body fluid, secretions, and excretions. Moreover, a majority of them reported always wearing gloves when they come in contact with blood or handling the patient’s mucosa (79.8%), dressing wounds (79.3%), disposing of stool or urine (77.9%), handling impaired patient skin (75.1%), performing parenteral injections of medications (72.8%), and drawing blood samples (68.5%).

In relation to hand hygiene, 75.1% of the participants always performed hand hygiene immediately after contacting blood, body fluid, secretion, excretion, and dirty substances. However, only 55.4% reported performing hand hygiene when they come in contact with patients and 44.6% reported performing hand hygiene after taking off gloves. While study participants always wear protective suits or a gown, 66.2% and protective eye patch or goggle (62.9%) when performing operations/procedures that might induce spraying of blood, body fluid, secretions, and excretions. About medical waste disposal, 79.8% of the participants reported always disposing of single-use needles, blades, and other sharp objects in a sharp disposal box after use.

Related to the factors that impact the level of knowledge about standard infection control precaution, the results presented in Table 5 revealed that the older HCWs (>34 years) were more likely to have good knowledge about standard precautions when compared with the younger HCWs (OR: 30.47, 95% CI: 8.34–111.25, *p* < 0.001). As well, the HCWs that received training on infection were 13.26 times more likely to have good knowledge than the ones who did not receive such training (OR: 13.3, 95% CI: 4.06–43.23, *p* < 0.001). However, surprisingly HCWs with more than 6 years of work experience were less likely to have good knowledge than those who had less work experience (OR: 0.14, 95% CI: 0.06–0.34, *p* < 0.001). While sex and previous exposure to infection while working did not have any statistically significant association with the level of knowledge about standard infection control precautions (*p* > 0.05).

With regard to the attitude of the HCWs towards standard infection control precautions, the results showed that there is no significant association with sex, age group, previous exposure to infection, and receiving training (*p* > 0.05). However, HCWs with more than 6 years of experience were more likely to have a positive attitude compared with the HCWs with less experience (OR: 5.46, 95% CI: 2.81–10.59, *p* < 0.001).

Regarding the practice of infection control standard precaution, the results showed that all characteristics of the participants are statistically associated with the level of practice of standard infection control precautions. HCWs who received training on infection control (OR: 3.54, 95% CI: 1.40–8.98, *p* < 0.01), who had more than 6 years of work experience (OR: 2.88, 95% CI: 1.38–5.99, *p* < 0.01) or were exposed to infection while working (OR: 2.45, 95% CI: 1.08–5.53, *p* < 0.05) were more likely to practice a good standard of infection control precautions. Sex and age were also associated with good practice. Female HCWs (OR: 0.22, 95% CI: 0.10–0.49, *p* < 0.001) and HCWs 35 years of age (OR: 0.34, 95% CI: 0.16–0.75, *p* < 0.01) were less likely to practice good standards compared with their counterparts.

The results in Table 6 show that HCWs having less working experience (1 to 6 years compared with more than 6 years) had a significantly higher mean knowledge score (mean difference: 0.5, 95% CI: 0.04–1.5, *p* < 0.05). In addition, the mean knowledge score was significantly higher among the male HCWs than the females (mean difference: 0.7, 95% CI: 0.02–1.5, *p* < 0.05).

In the case of attitude towards standard infection control precautions, we found that HCWs 35 years of age or more, female, having more than 6 years of working experience and being previously exposed to infection while working had a significantly higher attitude score compared with the HCWs less than 35 years of age, males, having up to 6 years of work experience and not being exposed to infection while working. Surprisingly, our results showed that the training had no statistically significant impact on the attitude of HCWs toward infection control standard precautions (*p* > 0.05).

In case of the practice of standard infection control precautions, we found that variables such as being younger (<34 years), male, having more experience (>6 years) and receiving training on infection control were associated with significantly higher mean practice scores when compared with their counterparts (*p* < 0.05).

## 4. Discussion

We assessed the KAP of standard infection control precautions among HCWs in Qassim, Saudi Arabia. The findings of the study suggest that more than 60.0% of HCWs practicing in Qassim Medical City University had good (≥80.0% correct response) knowledge, attitude, and practice. Over 34 years of age and receiving training were significantly associated with having good knowledge while >6 years of work experience was the only significant predictor of having a positive attitude. Having >6 years of work experience, previous exposure to HAIs and training were the significant predictors of good practice. On the other hand, female sex and older age were negatively associated with knowledge. Knowledge is essential to develop a positive attitude; therefore, it is a key instigator to bring a positive change in practice [22,23]. Evidence suggests that knowledge and positive attitudes are associated with improved compliance with infection control standard precautions among HCWs [32]. Here we report the status of KAP regarding standard precautions of infection control among the HCWs in the medical city of the Qassim University, KSA.

We found that just over two-thirds of the study participants had good knowledge (provided at least 80% correct answer) about infection control standard precautions. However, this rate is still higher than the rate reported by studies conducted in a hospital in Northern Cyprus [32], among nursing students in Jordon (49.64%) [33], and among the dental faculty members and students (3rd–5th year) in Riyadh, KSA (49–49.6%) [34]. Gaps in the knowledge of standard precautions among HCWs were also evident in studies conducted in Iran [35] and Nigeria [36]. This gap in knowledge among HCWs necessitates more emphasis on infection control standard precautions in academic and continued professional development training curriculums.

Despite having an average level of knowledge, most of the participants answered correctly to the knowledge statements related to using standard precautions for all patients regardless of their diagnosis, isolation precaution, and performing hand hygiene after contact with the patient’s environment, and before and after patient care. Moreover, most of them correctly answered the knowledge statements related to wearing and changing gloves for each patient. These findings are consistent with studies conducted in Nigeria [37] and India [38].

With regard to the attitude, our study found that the proportions of HCWs with a positive attitude (≥80% score) were 61.5%, which is considerably higher compared with studies conducted in Jordon [33] and Iran [39], but lower than the proportion reported in Ethiopia (64.2%) [40], and among the primary care professionals in Abha, KSA (88.2%) [41]. This difference between our study and the study conducted in the KSA is because of using a different classification system, such as any score ≥60% were classified as a positive attitude in the Abha study, while the cutoff point for a positive attitude in our study was 80%.

The majority of the HCWs had a positive attitude toward washing hands before and after any intervention with patients (91.5%); disinfection of medical equipment (85.0%); adhering to standard and contact precautions (78.4%) and believed that standard precautions prevent the spread of infections (87.8%). This is probably due to the fact that these activities became routine practice, which probably was reinforced by a positive institution culture and policies, introduced by the Ministry of Health, KSA on infection control.

Our study found that 73.3% HCWs had a good practice (≥80% score). This rate is higher compared with the findings from studies conducted in Vietnam (46.1%) [42], Northern Cyprus (30.9%) [32], Ethiopia (60.2%) [43], Iran (42%) [39] and Singapore (66.3%) [44], but lower than the rate reported among nurses in India (91%) [45]. These differences in the level of practice of infection control standard precautions in different countries may be due to the differences in education, training, organizational culture, policies, the presence of infection control guidelines and monitoring of its implementation.

Our logistic regression analysis results suggest that older (≥35 years) HCWs were more likely to have good knowledge compared with the younger HCWs (<35 years). This is in contrast with the findings reported in Cyprus [32] and in Egypt [46]. These studies reported that younger HCWs (<34 years) were more likely to have good knowledge. However, another study reported no association between age and knowledge in Ethiopia [47]. Our results also suggest that HCWs who received training on infection control standard precautions were 13.3 times more likely to have good knowledge than the HCWs without the training. This indicates the importance of training in refreshing and updating the HCWs’ knowledge on infection control standard precautions. Likewise, Elliott et al., argued that intensive teaching and self-learning can improve the knowledge of HCWs in preventing sharp injuries [48]. Surprisingly, we found that those with 6 years or more work experience were less likely to have good knowledge than the less experienced HCWs. Perhaps recent academic programs provide greater emphasis on topics about infection control or infection control guidelines developed recently by the health-care facility. Therefore, lower knowledge among more experienced HCWs may be partly related to lack training on infection control. Similarly, a study in the UK reported that current medical students demonstrated better knowledge of needlestick injuries than the previous cohort [48]. Regarding the association between sex and level of knowledge, we found no evidence of association. In contrast, a study among nurses in Iran found a significant association between sex and knowledge of infection control [49].

Our results also showed that sociodemographic variables such as age, sex, and training were not associated with attitude towards infection control standard precautions. This is in agreement with studies conducted in Nigeria [37], Turkey [50], and the Eastern province of the KSA [51]. On the contrary, we found that HCWs having more than 6 years of experience were more likely to have a positive attitude when compared with the less experienced ones. This denotes that experience is an important influencer of attitude. While a positive attitude is highly correlated with good practice and therefore is an important public health issue because these prevent the spread of infection from the health-care facilities [52].

Regarding the practice of infection control standard precautions, our results showed that all characteristics of the participants such as age, sex, experience, and training were significantly associated with good practices. This is consistent with a study conducted by AlKhaldi et al., who reported that good practice was significantly associated with years of experience and training in infection control [41]. A similar study in Korea reported that sex, work experience, age and training courses are significantly associated with practice [53]. Therefore, it is important that health-care facilities organize regular training programs on infection control standard precautions for the HCWs to refresh and update their knowledge and promote a positive attitude and good practice.

This study inherits some limitations. Our study findings are based on the data collected from a single health-care center, which might limit the generalization of the study findings. However, the Qassim University Medical City is a tertiary level hospital including 278 HCWs; which is one of the largest hospitals in the region. Additionally, this study utilized self-reported data and therefore we cannot rule out the possibility of information bias. Future studies incorporating observational data and documentary analysis are necessary to investigate what is happening in real-world practice. Despite these limitations, ours is the first study reporting KAP on standard precautions of infection control among HCWs in Qassim, KSA. The evidence generated from this study will inform policy and guide service providers to improve KAP of HCWs as well as to reduce the burden of HAIs in the KSA.

## 5. Conclusions

Having good knowledge, attitude, and practice of infection control standard precautions are vital to prevent the spread of infections from health-care facilities. Our research highlighted the gaps in KAP of the HCWs practicing in a teaching hospital in Qassim, the KSA. The duration of experience was negatively associated with knowledge which might indicate that older academic programs did not adequately cover topics on infection control in health-care facilities. We further found that receiving training on infection control standard precautions is positively associated with good knowledge and practice. Therefore, arranging training programs for HCWs might be useful in refreshing and improving their knowledge of infection control standard precautions and is also expected to facilitate positive attitude and practice.

## Figures and Tables

**Table 1:** Socio-demographics characteristics of HCWs, assessment of KAP of standard infection control precautions, Qassim University Hospital, KSA.

Total Characteristics	Count (%)
Sex Male Female	120 (56.3) 93 (43.7)
Age group 22–34 >35 years	143 (67.1) 70 (32.9)
Work experience 0–6 years >6 years	69 (32.4) 144 (67.6)
Exposed to infection while working	
Yes No	65 (30.5) 148 (69.5)
Received training on infection control	
Yes No	180 (84.5) 33 (15.5)

**Table 2:** Knowledge of HCWs on infection control standard precautions, Qassim University Hospital, KSA.

Knowledge Questions (Correct Response)	Correct Responses	
	Count	%
1. Standard precautions are used for the care of all patients regardless of their diagnosis and perceived infection status (Yes).	205	96.2
2. Isolation precaution is one of the elements in standard precaution (Yes).	162	76.1
3. Washing hands after contact with the patient’s environment is one of the elements in standard precaution (Yes).	204	95.8
4. Alcohol-based rubs are used after removing gloves (Yes).	122	57.3
5. Performing hand hygiene is required before and after patient care (Yes).	205	96.2
6. Hands should be washed with soap and water before and after handling potentially infectious materials irrespective of wearing gloves (Yes).	200	93.9
7. PPE is important in infection control because it acts as a barrier between infectious materials such as viral and bacterial contaminants and your skin, mouth, nose, or eyes (mucous membranes) (Yes).	166	77.9
8. Gloves must be worn every time during handling potentially infectious materials (Yes).	209	98.1
9. Gloves must be changed during patient care if you move hands from ‘contaminated body site’ to ‘clean body site’ (Yes).	204	95.8
10. Surgical masks can protect the nose and mouth when procedures and activities are likely to generate splashes or sprays of blood and body fluids (Yes).	177	83.1
11. The purpose of using a gown or apron is to protect clothes from splashes or sprays of blood and body fluids (Yes).	182	85.4
12. Removed all personal protective equipment (PPE) before leaving the patient’s environment (Yes).	130	61.0
13. Stationary, telephones kept in wards, and doorknobs can be sources of infections (Yes).	22	10.3
14. All linen from an infectious patient should be thrown in a red linen bag even when it is free from visible blood or body fluids (Yes).	97	45.5
15. Segregation of clinical and non-clinical waste is important for preventing the spread of infection (Yes).	205	96.2
16. Ampoules injection that has been used must be disposed of in the clinical waste bin (Yes).	79	37.1
17. Recapping of needles, in general, is not appropriate (Yes).	172	80.8
18. If you puncture hand with sharp instruments, you must report to the concerned authorities (Yes).	186	87.3
19. Puncture-proof containers should be used for disposal of sharps objects (Yes).	204	95.8
20. Mask must be placed on coughing patients to prevent potential dissemination of infectious respiratory secretions from the patient to others (Yes).	213	100.0
Overall level of knowledge Poor Moderate Good	13 56 144	6.1 26.3 67.6
Mean score (± SD)	15.7 (±2.7)	
Knowledge score range	9–20	

**Table 3:** Attitude of HCWs towards standard infection control precautions, Qassim University Hospital, KSA.

Items Used to Assess Attitude (Positive Attitude)	Positive Attitude	
	Count	%
1. Standard precaution is not easy to follow (strongly disagree).	82	38.5
2. Standard precautions prevent the spread of infections from patients to HCWs and vice versa (strongly agree).	187	87.8
3. Infectious diseases can be treated hence PPE are not required (strongly disagree).	117	54.9
4. Prefers to perform hand hygiene before and after any intervention with patients (strongly agree).	195	91.5
5. PPE can be used during emergencies (strongly agree).	94	44.1
6. Changing gloves is not necessary during procedures even if heavily contaminated (strongly disagree).	176	82.6
7. It is difficult to work wearing PPE (strongly disagree).	27	12.7
8. Healthcare providers should ensure the availability of adequate protective barriers (strongly agree).	145	68.1
9. HCWs should not use PPE because it may harm patients psychologically (strongly disagree).	129	60.6
10. Stationeries, telephones, and doorknobs are not sources of infections (strongly disagree).	158	74.2
11. Segregation of clinical and non-clinical waste is useful to prevent transmission of infections from one to another (strongly agree).	143	67.1
12. Adequate disinfection of medical equipment should be ensured by all HCWs (strongly agree)	181	85.0
13. Transmission of infectious organisms can be reduced by adhering to standard and contact precautions (strongly agree).	167	78.4
14. It is not logical to assume all patients contagious unless their infection has been confirmed (strongly disagree).	34	16.0
Overall level of attitude Negative Neutral Positive	9 73 131	4.2 34.3 61.5
Mean score (± SD)	55.5 (± 5.7)	
Range	35–65	

**Table 4:** Practice of standard infection control precaution among HCWs, Qassim University Hospital, KSA.

Items Assessed Practice	Good Practice	
	Count	%
1. Always performs hand hygiene when they come in contact with patients.	118	55.4
2. Always performs hand hygiene after taking off gloves.	95	44.6
3. Always washes hands immediately after contacting any blood, body fluid, secretion, excretion, or dirty substances.	160	75.1
4. Always wears gloves when drawing blood samples.	146	68.5
5. Always wears gloves when disposing of stool or urine.	166	77.9
6. Always wears gloves when handling impaired patient skin.	160	75.1
7. Always wears gloves when handling the patient’s mucosa.	170	79.8
8. Always wears gloves when handling saliva or sputum culture.	179	84.0
9. Always wears gloves when performing parenteral injections of medications.	155	72.8
10. Always wears gloves when dressing wounds.	169	79.3
11. Always wears gloves when they come in contact with blood.	170	79.8
12. Always wears mask when performing operations/procedures that might induce the spraying of blood, body fluid, secretions, or excretions.	175	82.2
13. Always wears a protective eye patch or goggle when performing operations/procedures that might induce spraying of blood, body fluid, secretions, or excretions.	134	62.9
14. Always wears protective suits or gown when performing operations/procedures that might induce spraying of blood, body fluid, secretions, or excretions.	141	66.2
15. Always dispose of needles, blades, or any other single use sharp objects in a sharp disposal container after use.	170	79.8
Overall level of practice Poor/moderate Good	57 156	26.8 73.2
Mean score (±SD)	14.2 (± 2.1)	
Range	5–15	

**Table 5:** Association between sociodemographic characteristics and level of knowledge, attitude, and practice of standard infection control precautions among HCWs, Qassim University Hospital, KSA.

Characteristics	Knowledge				Attitude				Practices			
	Poor/ Moderate (*n, %)*	Good (*n, %)*	*p*-Value	OR (95% CI)	Poor/ Moderate (*n, %)*	Positive (*n, %)*	*p*-Value	OR (95% CI)	Poor/ Moderate (*n, %)*	Good (*n, %)*	*p*-Value	OR (95% CI)
Sex												
Male	36 (30)	84 (70)		1	53 (44.2)	67 (55.8)		1	24 (20.0)	96 (80.0)		1
Female	33 (35.5)	60 (64.5)	0.459	1.37 (0.60–3.13)	29 (31.2)	64 (68.8)	0.353	1.38 (0.70–2.72)	33 (35.5)	60 (65.5)	0.000	0.22 (0.10–0.49)
Age groups												
22–34 years	65 (45.5)	78 (54.5)		1	54 (37.8)	89 (62.2)			35 (24.5)	108 (75.5)		1
>34 years	4 (5.7)	66 (94.3)	0.000	30.47 (8.34–111.25)	28 (40.0)	42 (60.0)	0.191	0.63 (0.32–1.26)	22 (31.4)	48 (68.6)	0.007	0.34 (0.16–0.75)
Work experience												
0 to 6 years	14 (20.3)	55 (79.7)		1	44 (63.8)	25 (36.2)			27 (39.1)	42 (60.9)		1
>6 years	55 (38.2)	89 (61.8)	0.000	0.14 (0.06–0.34)	38 (26.4)	107 (73.6)	0.000	5.46 (2.81–10.59)	30 (20.8)	114 (79.2)	0.005	2.88 (1.38–5.99)
Previously exposed to infection while working												
No	55 (37.2)	93 (62.8)		1	64 (43.2)	84 (56.8)		1	44 (29.7)	104 (70.3)		1
Yes	14 (21.5)	51 (78.5)	0.577	1.28 (0.54–3.04)	18 (27.7)	47 (72.3)	0.133	1.76 (0.84–3.66)	13 (20.0)	52 (80.0)	0.031	2.45 (1.08–5.53)
Received training in infection control												
No	21 (63.6)	12 (36.4)		1	17 (51.5)	16 (48.5)		1	17 (51.5)	16 (48.5)		1
Yes	69 (26.7)	132 (73.3)	0.000	13.26 (4.06–43.23)	65 (36.1)	115 (63.9)	0.563	1.28 (0.56–2.93)	40 (22.2)	140 (77.8)	0.008	3.54 (1.40–8.98)

**Table 6:** Differences between mean standard infection control standard precautions knowledge, attitude, and practice scores between different HCWs, Qassim University Hospital, KSA.

Socio-Demographic Characteristics	Knowledge			Attitude			Practice		
	Mean	Mean Difference (95% CI) *	*p*-Value	Mean	Mean Difference (95% CI) *	*p*-Value	Mean	Mean Difference (95% CI) *	*p*-Value
Age group									
22–34 years	16.2	0.7 (−0.04–1.5)	0.061	51.6			13.7		
>34 years	15.5			57.4	5.8 (4.3–7.3)	0.000	14.5	0.8 (0.3–1.5)	0.003
Sex									
Male	16.0	0.7 (0.02–1.5)	0.042	54.4			14.5	0.6 (0.1–1.2)	0.022
Female	15.3			56.9	2.5 (1.1–3.8)	0.001	13.9		
Work experience									
1–6 years	17.6	0.5 (0.04–1.5)	0.026	53.3			13.7		
>6 years	17.1			56.8	3.5(2.1–4.8)	0.000	14.5	0.8 (0.3–1.5)	0.003
Previously exposed to infection while working									
No	16.1	0.6 (0.2–1.4)	0.163	54.4			14.0		
Yes	15.5			57.5	3.1 (1.7–4.5)	0.000	14.8	0.8 (0.2–1.4)	0.010
Received training on infection control									
No	13.2			53.8			11.7	3.0 (2.4–3.7)	0.000
Yes	16.2	3 (1.9–3.8)	0.269	55.6	1.8 (0.7–4.3)	0.166	14.7		

* Independent sample *t*-test. Equal variances assumed.

## Data Availability

Data used in this study are available from the corresponding author on reasonable request.
